# Hyperglycemia-Induced Complete Left-Sided Hemiballismus Due to Uncontrolled Diabetes in a 70-Year-Old Female: A Case Report

**DOI:** 10.7759/cureus.53220

**Published:** 2024-01-30

**Authors:** Taylor F Faust, Julee Reitzel, Aftab Khan, Garrett M Cail, Raphael Quansah

**Affiliations:** 1 Department of Research, Alabama College of Osteopathic Medicine, Dothan, USA; 2 Department of Internal Medicine, Decatur Morgan Hospital, Decatur, USA

**Keywords:** diabetic ketoacidosis, diabete mellitus, putamen, basal ganglia disease, non-ketotic hyperglycemia hemichorea-hemiballismus syndrome

## Abstract

This report details the presentation of a 72-year-old female with left-sided continuous non-rhythmic involuntary movements persisting for two months. The movements affected the left side of her face, arm, and leg. The patient had a history of multiple hyperglycemic episodes and diabetic ketoacidosis. This report investigates the basal ganglia’s involvement in hemiballismus, a movement disorder possibly linked to the patient’s hyperglycemia. It discusses the complex management of hyperglycemia-induced hemiballismus and the need for more research to understand the underlying mechanism and optimal treatment strategies.

## Introduction

Diabetes mellitus is the eighth leading cause of death in the United States, yet more money is spent each year on diabetes than any other disease. With approximately 11% of the US population living with diabetes and an alarming increase in diabetic complications, the US spends an estimated 327 billion dollars each year managing this condition [1]. Poor management of blood glucose levels is associated with a higher risk of complications. Diabetic complications include but are not limited to stroke, myocardial infarction, end-stage renal disease, need for dialysis, amputations, retinopathy, and metabolic derangements. In this report, we explore a less common but well-described complication called nonketotic hyperglycemic hemichorea-hemiballism. 

Nonketotic hyperglycemic hemichorea-hemiballism, also known as "diabetic striatopathy," is a recognized cause of acquired hemichorea in adults, often associated with poorly controlled, long-standing type 2 diabetes [2]. Hemiballism involves unpredictable, violent limb movements, often stemming from various causes like cerebrovascular issues, traumatic brain injury, toxins, tumors, HIV, tuberculosis, paraneoplastic syndromes, neuromuscular disorders, vitamin deficiencies, and metabolic disorders [3].

Hemiballism is a hyperkinetic disorder involving the basal ganglia, a group of subcortical nuclei that coordinate movements. They control motor movements via projections onto the thalamus and upper motor neurons [4]. The striatum is comprised of the caudate and putamen and receives input from GABAergic, glutaminergic, and dopaminergic neurons [4]. The output nuclei (globus pallidus internus and substantia nigra pars reticulata) act as a "brake" on motor movements. The intrinsic nuclei include the pars compacta, a group of dopaminergic neurons projecting onto the striatum for finer motor control [4]. Two pathways govern motor control: the direct pathway, increasing activity by inhibiting the output nuclei, and the indirect pathway, inhibiting motor movements through thalamic inhibition. Hypokinetic disorders like Parkinson’s involve a constitutively active "brake," while hyperkinetic disorders like Huntington's, dystonia, and hemiballism result from a deactivated "brake."

Metabolic causes of hemiballism, while infrequent, are underestimated in clinical practice [5]. Hemi-chorea hemiballismus with nonketotic hyperglycemia is the most common, comprising 1% of recent chorea-ballism cases in a recent study [6]. The pathogenesis of nonketotic hyperglycemic hemichorea-hemiballism is not fully understood. Proposed mechanisms involve hyperglycemia impairing cerebral autoregulation, causing hypoperfusion, activating anaerobic metabolism, and depleting GABA in the basal ganglia [7]. This depletion results in hyperkinetic movements due to attenuated inhibition of the subthalamic nucleus by the medial globus pallidus [2]. In addition, hyperviscosity associated with hyperglycemia disrupts the blood-brain barrier, causing transient ischemia in striatal neurons [8]. The relationship between hemiballismus and hyperglycemia versus other metabolic byproducts is still unknown [9].

Age and female sex are identified as risk factors for this condition [10]. Patients typically exhibit continuous, involuntary movements primarily in the limbs and occasionally the face [11]. Diagnostic findings include nonketotic hyperglycemia, with CT revealing hyperdense areas and MRI displaying hyperintense T1-weighted signals in the basal ganglia [7]. Most patients experience chorea remission within days to weeks after achieving glycemic control, but some cases persist or recur after remission [12]. While glycemic control is the primary treatment, short-term antipsychotics may be necessary in some instances [12].

We present a case of a 72-year-old female with poorly controlled diabetes (hemoglobin A1c up to 16.5%) and multiple hospitalizations for hyperglycemia up to 897 mg/dL within a 17-month period. MRI revealed a right-sided T1 hyperintensity of the left putamen without signal intensity changes.

## Case presentation

A 72-year-old Caucasian female presented to our care with two months of left-sided continuous non-rhythmic involuntary movements. The movements include the left side of the face, left arm, and left leg. The onset of symptoms was insidious without change or resolution.

Seventeen months prior to the onset of her symptoms, she was hospitalized for hyperosmolar hyperglycemic coma due to uncontrolled diabetes mellitus with ketoacidosis. During that admission, her blood glucose was 897 mg/dL with an anion gap of 20 and a hemoglobin A1c of 14%.

The patient was put on diabetic ketoacidosis (DKA) protocol and an insulin regimen, which resulted in a full recovery. Fifteen months later, she returned to the hospital with a second episode of DKA and a glucose level of 888 mg/dL. She was treated, and DKA was resolved. The patient returned to the hospital to our care with multiple medical illnesses, including CHF exacerbation, uncontrolled diabetes, diverticulosis, renal mass, gastritis, chronic renal disease, and hypertension, and featured another episode of DKA with a glucose level of 512 mg/dL and an anion gap of 27. A magnetic resonance imaging (MRI) was completed during our care that featured diffuse cerebral atrophy with chronic microvascular ischemic changes affecting the basal ganglia, but no acute process.

Physical examination was significant for the left-sided face and upper and lower extremities, with hemi-chorea-like movements. The movements were increased in the upper extremity compared to the lower. Movements were suppressed during sleep, which was confirmed by the provider. Finger-to-nose maneuver featured mild dysmetria of the left side. Her pupils were bilaterally equal, round, and reactive to light, without nystagmus. Extraocular movements were intact. All cranial nerves were assessed and were found to be normal on examination. No motor and sensory deficits were observed. Coordination was intact. Muscle strength was 5/5 in all muscle groups. No atrophy or fasciculations were noted. 

On initial presentation, her vitals featured blood pressure of 78/40, pulse rate of 130, respiratory rate of 26, O_2_ at 99%, and temperature of 100.7 F˚. The initial laboratory workup (Table 1) revealed microcytic anemia, positive D-dimer, and renal insufficiency. At the time, the differential diagnosis included stroke, drug toxicity, Huntington's disease, and malignancy. There are no clinical features of a stroke, and it was excluded due to a lack of hemiparesis. There were no clinical manifestations or family history to support Huntington's disease, and she was beyond the age range known for Huntington’s disease emergence. The patient had no recent traumatic injury and was not taking antipsychotics or anticonvulsants.

**Table 1:** Initial laboratory work-up WBC: white blood cell count, RBC: red blood cell count, Hgb: hemoglobin, PT: prothrombin time, PO_2_: partial pressure of oxygen, BUN: blood urea nitrogen, AST: aspartate transferase, ALT: alanine aminotransferase, TSH: thyroid stimulating hormone

Initial laboratory work-up	Lab values	Reference ranges
Hematology		
WBC	26.16 x 1000/UL	(4.8-10.8 x 1000/UL)
RBC	(3.24 x MIL) g/dL	(4.2-5.4 x MIL) g/dL
Hgb	8.2 g/dL	(12-15.0 g/dL)
Hematocrit	27.70%	(37.0-47.0%)
Platelet count	291 x 1000/uL	(130-400 x 1000/uL)
Coagulation		
PT	16.5 seconds	(11-16 seconds)
D-dimer	5.24 ug/mL FEU	(0.0-0.52 ug/mL FEU)
Blood Gas		
PO_2_	124 mmHg	(60-100 mmHg)
Total hemoglobin	9.4 g/dL	(11.5-17.5 g/dL)
Chemistry		
Carbon dioxide	24 mmol/L	(25-35 mmol/L)
Sodium	144 mmol/L	(136-145 mmol/L)
Potassium	4.0 mmol/L	(3.5-5.1 mmol/L)
Chloride	103 mmol/L	(98-107 mmol/L)
BUN	61 mg/dL	(8-22 mg/dL)
Creatinine	3.1 mg/dL	(0.5-0.9 mg/dL)
Glucose	438 mg/dL	(70-104 mg/dL)
Calcium	8.5 mg/dL	(8.8-10.2 mg/dL)
Albumin	2.9 g/dL	(3.5-5.0 g/dL)
AST	11 U/L	(10-30 U/L)
ALT	6 U/L	(10-36 U/L)
Alk. phosphatase	56 U/L	(32-104 U/L)
TSH	0.58 uIU mL	(0.27-4.2 uIU mL)

**Figure 1:**
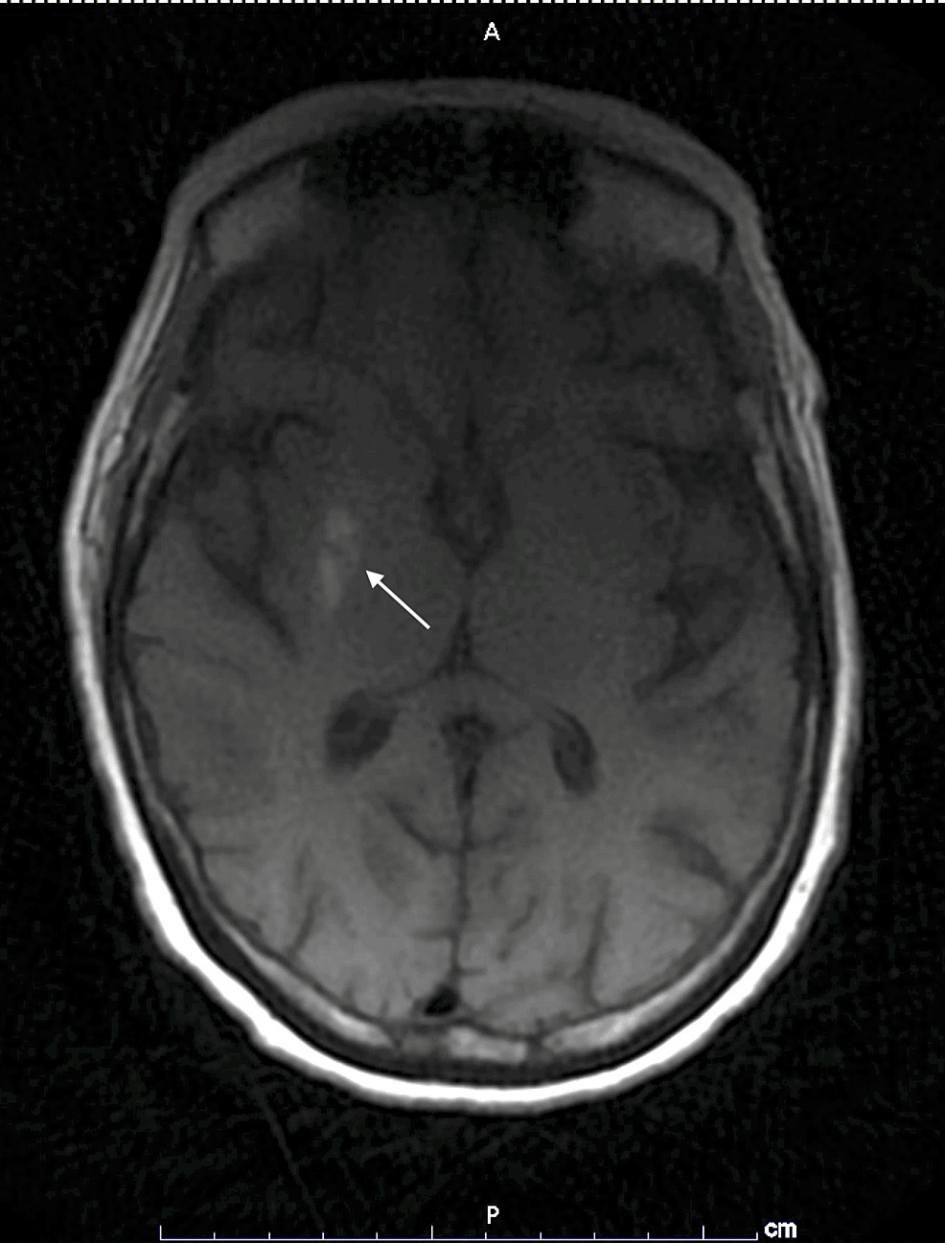
Brain MRI (sagittal): asymmetric T1 hyper-intensity of the right putamen (white arrow) A: anterior, P: posterior

The MRI was reviewed again, which revealed a right-sided T1 hyperintensity of the left putamen without signal intensity changes. This specific finding is known for hyperglycemia-induced hemichorea-hemiballismus syndrome (Figure 1). Our patient presented with a history of uncontrolled diabetes, with multiple hospitalizations of glucose levels of 888, 896, and 512 in a 17-month period and high A1c levels on multiple occasions, which most recently recorded 16.5% at the time of her being in care with us. 

Following her hospital course where her other medical ailments that were discussed earlier were managed, the patient was discharged on Novolin R FlexPen, five units before meals and at bedtime, and 20 units of insulin glargine nightly. The patient was initiated on ropinirole 0.25 mg twice a day during her hospital stay and that was continued at discharge. In addition, the patient was started on olanzapine 2.5 mg orally every night at bedtime for involuntary movements. The patient was subsequently sent to rehabilitation to aid in her recovery. The patient will follow up with her primary care provider.

## Discussion

The basal ganglia are a group of subcortical nuclei involved in motor control, motor learning, executive functions, behaviors, and emotions [13]. Multiple movement disorders, including chorea, dystonia, Huntington’s disease, Parkinson’s disease, tic disorder, and Tourette syndrome, have been associated with damage to the basal ganglia. In this report, we discuss the basal ganglia syndrome known as hemiballism. It is a rare hyperkinetic movement disorder that is characterized by involuntary, violent coarse, and wide-amplitude movements that involve the ipsilateral arm and leg [3]. This is the result of a disruption of the network within the basal ganglia and its associated communicating nuclei.

Our patient displayed left-sided continuous non-rhythmic involuntary movements of the left side of the face, left arm, and left leg consistent with hemiballismus. Given her history, we considered stroke, drug toxicity, movement disorders such as Huntington’s disease, and other metabolic/toxic causes. However, we ruled out Huntington’s and drug toxicity based on the workup and patient history. Subthalamic stroke remained in the differential based on the location of the lesions on MRI and exam findings. However, given the onset of symptoms in the setting of extreme elevations in blood glucose and lack of acute findings on MRI to suggest evolving stroke, we felt that her presentation was most consistent with metabolic causes of hemiballismus.

Nonketotic hyperglycemia-induced hemichorea-hemiballismus is a rare complication of poorly controlled diabetes [9]. While the exact pathogenesis of this disorder remains unclear, there is an apparent link between the extraordinary elevations in blood glucose and the onset of symptoms. In diabetes mellitus, persistently elevated blood glucose is associated with an increased risk of cardiovascular disease, stroke, chronic kidney disease, and other chronic complications. These associations are understood through accepted mechanisms such as the polyol pathway, non-enzymatic glycosylated end products, and dysfunction in vascular smooth muscle and endothelial cells. In nonketotic hyperglycemia-induced hemiballismus, some authors suggest that hyperglycemia may impair cerebral autoregulation [7]. Other literature has suggested that hyperviscosity associated with hyperglycemia may disrupt the blood-brain barrier leading to transient ischemia in striatal neurons [8].

Diagnosis of hyperglycemia-related hemiballismus involves CT or MRI, with CT showing unilateral hyperdense basal ganglia. However, MRI remains the most sensitive. In hemiballismus, dysfunction can occur in the subthalamic nucleus, globus pallidus, putamen, and caudate nucleus. Specific MRI features include T1-weighted hyperintensity and T2-weighted hypointensity in the contralateral putamen [14]. Diagnosis also relies on clinical observations, such as chorea and hemiballism, that worsen during stress and disappear during sleep [15]. Laboratory tests include serum glucose, HbA1c, and serum osmolality, with high levels indicative of diabetic striatopathy. 

Typically, patients that present with nonketotic hyperglycemia-induced hemichorea-hemiballismus are older Asian females; however, there are numerous reports that show that the presentation can vary and be present in younger individuals (50+), males, and across different ethnicities [15]. Patients typically present with unilateral chorea. Initial lab testing in the vast majority of the reported cases shows blood glucose levels >400 mg/dL [12]. However, the urinalysis is negative for ketones. MRI scans of these patients show hyperintense signaling in the basal ganglia on the contralateral side of the hemichorea. Treatment and resolution of this condition require aggressive glycemic control. If symptoms persist or are refractory, additional agents, such as anti-choreic drugs or dopamine D2 receptor antagonists, may be indicated and help with the resolution of the chorea.

Preventing nonketotic hyperglycemia-induced hemichorea-hemiballismus involves managing diabetes and controlling blood glucose levels. Hyperglycemia is defined as blood glucose levels exceeding 125 mg/dL fasting and 180 mg/dL postprandial. Symptoms of hyperglycemia include polyuria, polydipsia, and weight loss, progressing to neuropathies and other complications [16]. Failure to adequately achieve optimal glycemic control may result in a prolonged duration of hyperviscous blood that may result in further complications, such as strokes, as was reported in a particular patient [17]. In addition, it can lead to cerebral volume loss in both the gray and white matter volume that can be detected on imaging [18].

Hemichorea-hemiballismus resolves in the majority of cases after initiation of aggressive glycemic control [19]. The symptoms may resolve over a period of several months. Despite the aggressive management of our patient’s blood glucose levels, her symptoms did not improve during the hospital stay. This suggests that the changes in the striatum induced by hyperglycemia may not be entirely reversed through glucose correction alone and may require additional therapy. Such cases may be considered refractory and may require the use of additional agents. Specifically, anti-choreic medications have demonstrated marked clinical improvement [12]. In addition, drugs that block postsynaptic dopamine D2 receptors, such as haloperidol or risperidone, may be indicated [15]. Clonazepam, tetrabenazine, and tipride can be considered for use if the patient does not respond [12]. Recent studies have also suggested improvement with topiramate, mainly attributed to its GABAergic properties [20].

## Conclusions

This case study highlights an instance of complete left-sided hemiballismus attributed to hyperglycemia. To enhance future diagnostic approaches, it is recommended to assess blood glucose and A1c levels promptly upon arrival of patients exhibiting hemiballismus and other hyperkinesia-like movements. Specifically, in cases involving a history of uncontrolled diabetes marked by recurrent episodes of diabetic ketoacidosis that require hospitalizations, comprehensive brain imaging is warranted, specifically CT and MRI scanning. 

Key observations on CT and MRI revealed hyperintensities in the basal ganglia and its associated structures. Due to the rarity of this disease's presentation with the common metabolic dysfunction of hyperglycemic episodes and diabetes, the early identification of glucose levels is paramount to addressing the hyperglycemic process and mitigating the possible occurrence of hemiballismus. In addition, more research is needed to identify the early intervention protocols and to explore treatment options aimed at alleviating hemiballismus and other hyperkinetic movements as a possible outcome.
